# Heart Failure Beyond the Heart: Digestive-System Involvement and Its Diagnostic and Prognostic Implications—A Systematic Review

**DOI:** 10.3390/diagnostics16203342

**Published:** 2026-10-14

**Authors:** Dragoș Forțofoiu, Ioana-Gabriela Dragne, Veronica Gheorman, Amelia-Valentina Genunche-Dumitrescu, Cristin-Constantin Vere, Dan-Nicolae Florescu

**Affiliations:** 1 Doctoral School, University of Medicine and Pharmacy of Craiova, 200349 Craiova, Romania; dragos.fortofoiu@umfcv.ro (D.F.); gabriela.dragne@umfcv.ro (I.-G.D.); 2 Department of Semiology, University of Medicine and Pharmacy of Craiova, 200349 Craiova, Romania; 3 Department of Internal Medicine, University of Medicine and Pharmacy of Craiova, 200349 Craiova, Romania; amelia_genunche@yahoo.com; 4 Department of Gastroenterology, University of Medicine and Pharmacy of Craiova, 200349 Craiova, Romania; cristin.vere@umfcv.ro (C.-C.V.); dan.florescu@umfcv.ro (D.-N.F.); 5 Research Center of Gastroenterology and Hepatology, University of Medicine and Pharmacy of Craiova, 200638 Craiova, Romania

**Keywords:** heart failure, digestive-system, cardio-hepatic syndrome, splanchnic congestion, intestinal oedema, gut barrier, pancreas, malnutrition, cardio-gastrointestinal syndrome

## Abstract

**Background/Objectives:** Heart failure (HF) is a systemic syndrome in which congestion, hypoperfusion, inflammation and neurohormonal activation may affect the liver, splanchnic circulation, intestinal barrier, pancreas, biliary system and nutritional state. This systematic review synthesised clinical evidence on digestive-system involvement in adults with established HF and examined its diagnostic and prognostic implications within a cautious cardio-gastrointestinal syndrome (CGIS) framework. **Methods:** The structured synthesis was based on an audited historical review register. The original project record reported 815 records before deduplication, although source-specific yields and the citation-level deduplication trail could not be reconstructed from the preserved files. A reproducibility–validation rerun was executed on 8 August 2026 in PubMed/MEDLINE, Scopus and Web of Science Core Collection, targeting literature through 1 July 2026; exact day-level cutoff reproducibility differed by database. The rerun recovered all ten benchmark reference-set reports but was not used to redefine the historical corpus and does not establish search exhaustiveness. The final 29-report register was re-audited for eligibility, design, analysed sample, principal findings and cohort overlap. Owing to substantial heterogeneity, findings were synthesised narratively. **Results:** 29 eligible reports represented approximately 26 cohort families. Hepatic and splanchnic involvement was the most mature evidence cluster, followed by intestinal congestion/barrier dysfunction. Pancreatic, biliary, microbiota and nutritional findings were less independently replicated. Liver biochemistry, composite hepatic scores and venous Doppler phenotypes showed marker-specific prognostic associations. Intestinal oedema was associated with impaired oral diuretic response. Direct pancreatic exocrine-insufficiency evidence was limited to faecal-elastase assessment. **Conclusions:** Digestive involvement in HF is clinically coherent but unevenly evidenced. CGIS is best regarded as a hypothesis-generating framework rather than a validated diagnostic entity, and prospective standardised studies are required before organ-specific biomarkers or imaging thresholds enter routine algorithms.

## 1. Introduction

Heart failure (HF) is a systemic clinical syndrome whose morbidity cannot be explained by impaired cardiac output alone. Recurrent congestion, neurohormonal activation, endothelial dysfunction, inflammation and progressive frailty generate a multiorgan phenotype that contributes to hospitalisation and death [1]. Although cardiorenal interactions are embedded in contemporary HF assessment, digestive-system involvement remains comparatively fragmented across cardio-hepatic, intestinal, pancreatic, nutritional and microbiome-focused literatures.

Two haemodynamic processes are central to digestive injury in HF: reduced forward flow, which compromises splanchnic perfusion, and elevated systemic venous pressure, which causes portal, hepatic and intestinal congestion. Landmark physiological studies documented reduced intestinal perfusion, bowel-wall thickening, increased permeability, bacterial translocation and impaired absorption in chronic HF [2,3,4]. In parallel, hepatic congestion and hypoxic injury produce cholestatic or hepatocellular biochemical patterns whose prognostic relevance has been repeatedly demonstrated [5].

Beyond the liver and intestine, smaller bodies of evidence suggest pancreatic exocrine dysfunction, altered pancreatic enzymes, gallbladder-wall oedema and clinically important malnutrition or sarcopenia. Pancreatic involvement is biologically plausible because the organ is exposed to the same low-flow and venous-congestion milieu as the bowel and liver [6]. Dysbiosis and gut-derived metabolites provide an additional route through which local intestinal injury could amplify systemic inflammation and HF progression [7].

These observations are usually considered separately. The term cardio-intestinal syndrome generally focuses on the heart–gut axis, whereas cardio-hepatic syndrome isolates hepatic consequences. Neither label adequately captures the full digestive phenotype arising downstream of HF. A broader construct—Cardio-Gastrointestinal Syndrome (CGIS)—could be clinically useful if it integrates objective organ injury, congestion, impaired absorption, microbial signalling and nutritional decline without implying that all components are equally validated.

Accordingly, this systematic review aimed to synthesise clinical evidence on the effects of established HF on the digestive-system, compare the maturity of evidence across organ domains, identify diagnostic and prognostic implications, and evaluate whether the findings support a cautious, testable CGIS framework.

## 2. Materials and Methods

### 2.1. Design and Reporting Framework

This systematic review was reported with reference to the PRISMA 2020 statement and the PRISMA-S extension for literature searches [8,9]. Limitations in the reproducibility of the historical search and selection audit trail are explicitly reported below. PRISMA 2020 and PRISMA-S are reporting frameworks, and the present audit should not be interpreted as establishing full reproducibility of an incompletely preserved historical search. We therefore distinguish the systematic methods documented for the review from the degree to which the historical audit trail can now be reconstructed. A formal protocol (version 1.0) for this review was finalised on 21 July 2026 after the review project had already begun; accordingly, the work was not prospectively preregistered. The project history records a subsequent retrospective OSF-registration step, but a persistent review-specific identifier is not present in the retained audit files and is therefore not asserted in this manuscript. The protocol defined the direction of interest as HF leading to digestive-system involvement; studies in which digestive disease was considered only as a precursor or risk factor for incident HF were not eligible for the primary synthesis. Protocol amendments and audit-driven clarifications were documented rather than backdated.

### 2.2. Review Question and Eligibility Criteria

The review question was as follows: in adults with acute or chronic HF, which objective digestive-system abnormalities are associated with HF, and what diagnostic, prognostic or therapeutic implications do they have? Eligible studies enrolled adults with established acute, decompensated, chronic, advanced or phenotype-specific HF and reported at least one objective hepatic, splanchnic, gastrointestinal, intestinal-barrier, microbiota, pancreatic, biliary, nutritional or multiorgan-congestion outcome. Nutritional, sarcopenia and multiorgan-congestion studies were retained as secondary or downstream CGIS evidence and were not interpreted as direct proof of digestive-organ injury. Observational cohorts, registry analyses, imaging studies, biomarker studies, clinical interventions and relevant clinical-trial sub-analyses were eligible.

Reviews, meta-analyses, editorials, conference abstracts, protocols, case reports, paediatric studies, animal or in vitro research, and studies lacking an objective digestive or CGIS-related outcome were excluded from the primary synthesis. Contextual reviews and landmark studies outside the final included set were used only to explain mechanisms and historical development. Because the retained files do not document an a priori publication-year restriction, none is asserted in this revision. The 2016–2025 interval is reported only as the observed publication range of the verified 29-report register. Older landmark reports cited in the Introduction were not retrospectively promoted into the audited corpus. Given the incomplete historical selection trail, their absence from the verified register cannot be interpreted as proof that they failed the eligibility criteria; this is treated as a limitation of corpus auditability rather than resolved retrospectively by selective inclusion.

### 2.3. Information Sources and Search Strategy

The retained project files document a historical multi-source search process, but they do not preserve a source-wise audit trail sufficient to support database-specific yields for the original 815-record aggregate. The structured synthesis therefore retains the historical aggregate flow only and does not claim unrecoverable source-level counts. To test reproducibility of the search concepts without redefining the included corpus, a validation rerun was executed on 8 August 2026 in PubMed/MEDLINE, Scopus and Web of Science Core Collection, targeting literature from database inception through 1 July 2026. The PubMed strategy encoded this cutoff directly. In the retained supplementary audit, the Scopus query used a publication-year filter rather than a day-level date limit, and the displayed Web of Science syntax does not itself encode the cutoff; these database-specific reproducibility limitations are stated in Supplementary Table S1. The validation strategies combined HF terms with objective digestive-system concepts spanning intestinal congestion and function, microbiota/barrier markers, hepatic and splanchnic congestion, pancreatic and biliary involvement, and VExUS-related venous phenotyping. The rerun retrieved 3660 PubMed records, 9122 Scopus records and 4269 Web of Science records. Each strategy recovered the same ten known reports used as a benchmark reference set. The retained files do not preserve a prospectively documented selection rule for this set; accordingly, the reports are not described as prespecified or as a statistically representative sample. Their recovery is interpreted only as a sensitivity/retrievability check for known reports and does not demonstrate completeness or exhaustive recall. These validation yields are reported separately in Supplementary Table S1 and were not substituted into the historical flow, screened as a new corpus, or used to redefine the 29-report synthesis. Embase was not available for the validation rerun.

The historical project record reported 815 records, 315 duplicate records removed, 500 records screened and 150 full-text reports assessed. Because the source-wise raw exports, exact original search dates, citation-level duplicate log and complete row-level full-text exclusion register were not preserved in a form that could be independently reconstructed, these values are reported explicitly as historical aggregate counts rather than newly verified database-level counts. In particular, individual reasons for the 121 full-text exclusions are not retrospectively reconstructed, because assigning reasons without a preserved row-level register would create an unverifiable selection record. The audit therefore focuses on the verified 29-report structured synthesis and on transparent identification for which elements are verified, historically reported, or no longer reproducible from retained materials.

### 2.4. Study Selection and Data Extraction

The retained review documentation states that title/abstract and full-text eligibility were assessed by two reviewers with consensus resolution, but the complete reviewer-level decision trail for the historical 815-record flow is not preserved in the audit files. The final 29-report register was therefore re-audited at publication level for eligibility, bibliographic identity, actual analysis design, analysed sample, principal finding and cohort overlap. For each eligible publication, the audit dataset captured publication year, actual analysis design, analysed sample, HF setting, digestive domain, measurement or biomarker, principal result, clinical significance and role in the proposed CGIS framework. Potential cohort overlap was assessed using centre or institution, investigators and authorship, recruitment interval, baseline sample size, HF phenotype, inclusion criteria, participant characteristics, and any shared study programme, trial or registry. Complete overlap was assigned when reports clearly analysed the same participant set; substantial overlap when a large shared population was strongly indicated; possible overlap when provenance suggested shared participants but the degree could not be quantified; and independent cohort when no material overlap was identified. Linked reports were retained when they contributed distinct outcomes, but independence-sensitive interpretation was based on cohort families rather than publication counts. Twenty-nine eligible publications or reports, representing approximately 26 cohort families, formed the verified structured clinical synthesis. The study–report linkage, cohort-family identifiers and assessment of publication independence are provided in Supplementary Table S2.

### 2.5. Methodological Appraisal

Methodological appraisal was audited at the level of the actual analysis question rather than the label of the parent study. Longitudinal and prognostic exposure analyses, including secondary analyses derived from randomised trials, were mapped to cohort-style appraisal; analytical cross-sectional and case-control components were mapped to design-appropriate JBI frameworks [10]; and the randomised controlled trial framework was reserved for the PACMAN-HF nutritional intervention analysis. Mixed-design publications were considered component-wise. The preserved project files did not permit a reliable reconstruction of the previously reported pooled item-level JBI totals across different instruments; those aggregate counts were therefore withdrawn rather than recreated. Supplementary Table S3 is the designated study-level appraisal record, reporting the actual analysis design, the design-appropriate JBI framework, a concise appraisal rationale and the principal methodological concern for each included report. Because the historical item-by-item checklist responses are not preserved, the revision does not retrospectively manufacture item-level JBI judgements. Appraisal findings were carried forward into domain-specific interpretation in the Results and Discussion. No publication was excluded solely on the basis of appraisal, and no global numerical quality score was calculated.

### 2.6. Synthesis

Clinical, methodological and measurement heterogeneity precluded a defensible pooled effect estimate. A structured narrative synthesis was therefore undertaken by digestive domain: intestinal congestion and barrier/function; microbiota; liver and splanchnic congestion; pancreas; biliary manifestations; nutrition, sarcopenia and cachexia; and multiorgan venous congestion. Direct organ-specific evidence was interpreted separately from secondary or downstream CGIS manifestations. Where primary reports provided verifiable effect estimates, clinically relevant HRs, ORs, confidence intervals or discrimination measures were reported selectively rather than treating statistical significance as evidence of clinical utility. Evidence maturity was assessed qualitatively according to independent replication, sample context, objectivity of measurement, consistency of association, methodological limitations and the degree of cohort overlap. Labels used in the evidence map were descriptive synthesis categories rather than formal certainty or guideline grades. “Most established” denoted repeated clinical associations across multiple reports with at least some independent replication; “emerging” denoted clinically coherent signals with limited replication or methodological heterogeneity; “limited”, “preliminary” and “exploratory” denoted sparse or single-report evidence; and “supportive” or “non-specific” denoted clinically relevant downstream or multiorgan findings without digestive-organ specificity.

## 3. Results

### 3.1. Study Selection and Evidence Distribution

**Figure 1:**
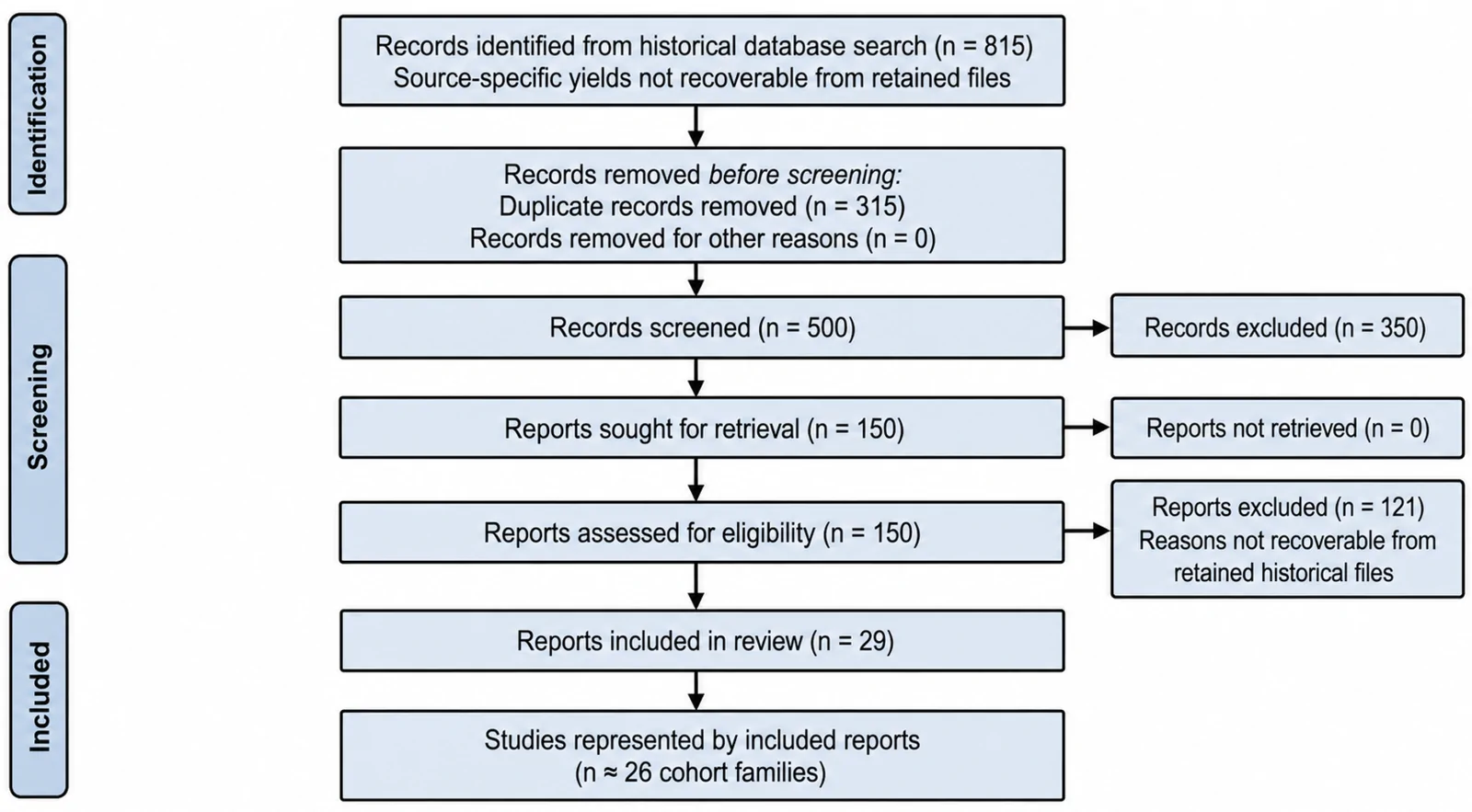
Historical aggregate study-selection flow retained in the project record. The counts are historical aggregate values; source-specific yields, the citation-level deduplication trail and individual full-text exclusion reasons could not be reconstructed from retained files and are not presented as newly verified PRISMA data.

The retained historical search record documented 815 records, with 315 duplicates removed and 500 records proceeding to title/abstract screening. Three hundred and fifty records were reported as excluded, leaving 150 full-text reports assessed. The verified structured register contains 29 eligible publications or reports. The preserved project files do not contain a complete row-level log for the remaining 121 full-text records; therefore, no retrospective reason-by-reason reconstruction is presented and the previously stated 117 contextual/4 insufficient-information breakdown is not reproduced as verified PRISMA exclusion data. Because several eligible publications arose from overlapping cohort programmes, the 29 reports represented approximately 26 cohort families. Figure 1 therefore presents a historical aggregate flow with explicit audit limitations. It should not be read as a newly reconstructed or fully reproducible source-level PRISMA flow.

**Figure 2:**
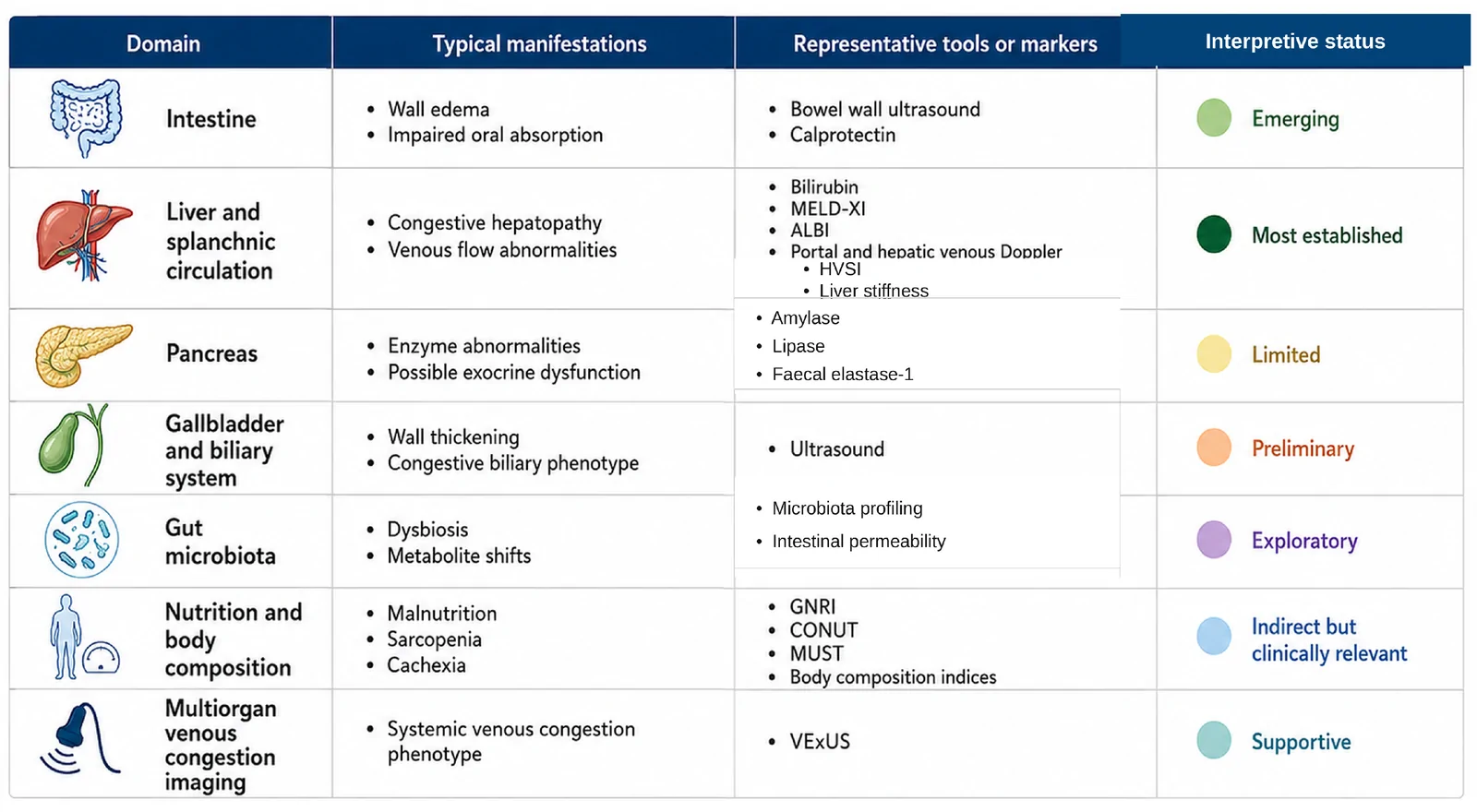
Evidence map of digestive-system manifestations in heart failure. Evidentiary labels are descriptive synthesis categories rather than formal certainty or guideline grades; they reflect replication, methodological consistency, objectivity of measurement and cohort independence.

The 29 included reports in the verified register were published between 2016 and 2025; this is an observed range and is not presented as an a priori publication-year restriction. Thirteen reports addressed hepatic, cardio-hepatic or splanchnic congestion and represented approximately 11 cohort families; five addressed intestinal congestion, barrier dysfunction or gastrointestinal function; four pancreatic reports represented three cohort families; and four reports addressed malnutrition, sarcopenia or nutritional intervention. One report each addressed microbiota, VExUS-based multiorgan congestion and biliary congestion. Each report was assigned to one primary domain for counting purposes, with secondary multidomain findings described narratively rather than double-counted. This distribution demonstrates both the maturity of the cardio-hepatic literature and the relative scarcity of direct, independently replicated evidence for other digestive organs. The relative maturity, representative measurements and interpretive status of each domain are summarised in Figure 2 and Table 1.

### 3.2. Characteristics of Included Studies

The characteristics of the 29 included reports are summarised in Table 2. The evidence base comprised predominantly observational studies, including prospective and retrospective cohorts, cross-sectional investigations, registry-based analyses and secondary analyses of clinical trials. Study populations varied considerably in terms of heart failure setting, encompassing acute decompensated heart failure, chronic heart failure, heart failure with reduced or preserved ejection fraction and advanced disease. Sample sizes ranged from small single-centre cohorts to large multicentre and database-derived populations. The studies also differed substantially in their diagnostic approaches, including laboratory biomarkers, abdominal and venous Doppler ultrasonography, gastrointestinal functional assessments and nutritional evaluations. Several publications originated from overlapping patient cohorts, particularly the Fukushima imaging programme and the pancreatic studies by Hiki et al., limiting the number of independent observations.

### 3.3. Intestinal Congestion, Barrier Dysfunction and Gastrointestinal Function

Five reports examined intestinal congestion, gastrointestinal function, inflammatory or permeability markers, or breath-test phenotypes [12,13,14,15,16]. Valentova et al. associated bowel-wall thickening with right-ventricular dysfunction, appetite loss, inflammation and cachexia, supporting a venous-congestion pathway while not establishing causality [12]. In Mollar et al., each 1000-unit increase in hydrogen breath-test AUC was associated with all-cause death/all-cause hospitalisation (HR 1.21, 95% CI 1.04–1.40) and death/HF hospitalisation (HR 1.20, 95% CI 1.03–1.40), whereas methane AUC was not associated with these endpoints [13]. Breath testing remained an indirect surrogate rather than a validated SIBO or microbiome phenotype.

Ikeda et al. reported that greater colon-wall thickness was associated with higher diuretic requirements and poorer response to oral, but not intravenous, loop diuretics [14]. Abdominal ultrasonography was performed under clinically stable conditions after initial HF treatment, so bowel-wall oedema was not demonstrated to precede the observed oral diuretic response. The result is compatible with impaired absorption but does not establish causality or a treatment-selection rule. In older patients with acute HF, Covino et al. found that faecal calprotectin above 50 micrograms/g, but not serum or faecal zonulin, was independently associated with the 90-day composite of death or acute-HF rehospitalisation [15]. Hao et al. documented gastrointestinal wall thickening, reduced motility and altered splanchnic or hepatic venous measures in acute HF, but did not establish prospective diagnostic thresholds or prognostic validation [16]. Across this domain, small samples, single-centre designs and uncertain temporality limit confidence in causal or clinical-utility claims.

### 3.4. Microbiota and Inflammatory–Microbial Signalling

Pasini et al. described pathogenic gut flora, increased intestinal permeability and associations with HF severity, estimated right-atrial pressure and C-reactive protein [11]. As the sole microbiota-focused primary report in the structured set, and given its cross-sectional case–control design, the study supports biological disturbance of the intestinal ecosystem but cannot establish temporality, independent replication or a reproducible diagnostic signature. TMAO literature informed the mechanistic framework, but the incident-HF cohort previously labelled Tang 2024 did not satisfy the established-HF population criterion and was therefore not counted in the primary synthesis.

### 3.5. Liver, Cardio-Hepatic Dysfunction and Splanchnic Congestion

The 13-report hepatic and splanchnic cluster was the most extensive and represented approximately 11 cohort families [17,18,19,20,21,22,23,24,25,26,27,28,29]. Analyses of acute-HF cohorts and trial-derived populations showed that liver-test abnormalities were common, dynamic and associated with clinical severity, although prognostic performance was marker-specific [17,18,22,29]. In ASCEND-HF, bilirubin retained independent prognostic associations, whereas aminotransferases were not uniformly independently associated with outcomes [18]. The collective pattern is compatible with both congestion-related cholestasis and low-flow or hypoxic injury, with the relative contribution varying by HF phenotype and timing of measurement. Interpretation is nevertheless limited by residual confounding, complete-case analyses in several datasets and heterogeneity in timing of biomarker measurement.

Composite indices broadened risk stratification beyond isolated liver enzymes. MELD-XI was associated with short- and longer-term adverse outcomes in large HFpEF and chronic-HF cohorts [23,25], while Curcio et al. reported associations between MELD and advanced-HF severity and outcomes [28]. These scores combine hepatic and renal information and should not be interpreted as liver-specific measures. In the comparative acute-HF analysis by Ali et al., ALBI, MELD-Alb, FIB-4 and FIB-5 remained statistically significant as continuous variables after adjustment, whereas MELD-XI did not; ALBI showed the highest reported discrimination (AUC 0.935) [27]. This single-centre estimate requires external validation and should not be interpreted as proof of incremental clinical utility. In TOPCAT HFpEF participants, bilirubin and alkaline phosphatase, but not AST or ALT, were independently associated with outcomes [24].

Imaging reports evaluated the venous compartment directly. Portal vein pulsatility was associated with right-ventricular dysfunction and mortality, but its addition to the EVEREST score changed the C-index only from 0.813 to 0.818 [19]. Liver stiffness, coeliac artery flow, hepatic-vein waveform patterns and the hepatic venous stasis index were associated with haemodynamics and clinical risk [20,21,26]. For example, the reversed hepatic V-wave pattern in Sugawara et al. was associated with cardiac events (adjusted HR 4.90, 95% CI 2.23–10.74) [21]. The Yoshihisa, Sugawara and Ohara publications arose from a nested, overlapping Fukushima imaging programme and therefore provide complementary analyses rather than three independent external replications. Acquisition protocols, thresholds and incremental prognostic performance require validation in independent multi-centre cohorts before routine implementation.

### 3.6. Pancreatic Involvement

Four pancreatic publications represented three cohort families [30,31,32,33]. Xia et al. provided the only direct evidence of pancreatic exocrine insufficiency, with faecal elastase-1 below 200 micrograms/g in 56.7% of patients and associations with appetite loss and HF severity [30]. Its selected supplementation subgroup was small and should not be interpreted as definitive intervention evidence. Vijver et al. observed modest increases in serum amylase and lipase during acute-HF hospitalisation, correlated with NT-proBNP changes, but baseline values were generally normal and the study did not directly test exocrine insufficiency [31]. The two Hiki publications arose from a substantially overlapping ADHF cohort: low admission lipase was independently associated with death or ADHF readmission (adjusted HR 1.96, 95% CI 1.16–3.32), while the complementary report compared enzyme levels with controls and described serial increases after clinical improvement [32,33]. These reports should not be treated as independent replication, and serum enzyme concentrations do not establish pancreatic exocrine insufficiency.

### 3.7. Biliary and Multiorgan Venous-Congestion Imaging

Sakamoto et al. evaluated gallbladder-wall thickness as an organ-congestion marker [34]. Gallbladder-wall thickness increased across HF stages, but Stage B participants were included; the prognostic signal should therefore be interpreted in the separable Stage C/D subgroup, in which greater thickness was associated with HF hospitalisation. The finding remains a single-report signal and does not justify a validated biliary phenotype. Anastasiou et al. evaluated multiorgan congestion with the venous excess ultrasound score in 290 patients with acute HF [38]. VExUS grade 3 was associated with in-hospital mortality (OR 8.03, 95% CI 2.25–28.61), and adding VExUS to GWTG-HF improved model fit (Δχ^2^ + 8.44, *p* = 0.03). This remains single-cohort evidence without external validation, and VExUS integrates renal and systemic venous components; it should therefore be regarded as a supportive multiorgan congestion phenotype rather than a digestive-organ diagnostic test.

### 3.8. Malnutrition, Sarcopenia and Cachexia

Four reports addressed nutritional risk, sarcopenia or nutritional intervention [35,36,37,39]. In the HELP-HF analysis, GNRI-defined malnutrition was present in 35.1% of the analysed sample and was independently associated with all-cause mortality (adjusted HR 2.03, 95% CI 1.43–2.89), but 55.6% of the parent registry lacked the data required for GNRI calculation, creating substantial complete-case selection concerns [35]. Outpatient studies confirmed high burdens of malnutrition, cachexia, frailty and sarcopenia [36,37]. In PACMAN-HF, nutritional intervention improved nutritional status and six-minute walk distance, but the composite of death or first HF hospitalisation was not significantly reduced (HR 0.34, 95% CI 0.11–1.09; *p* = 0.072) [39]. These reports contain no direct digestive-organ measurement and were therefore interpreted as secondary or downstream CGIS evidence. Their outcomes are multifactorial and may reflect congestion, anorexia, inflammation, immobility and systemic catabolism, as well as possible malabsorption.

### 3.9. Methodological Appraisal and Evidence Limitations

After audit of the actual analysis question, methodological appraisal was re-mapped at analysis level rather than according to the label of the parent study. Objective laboratory or imaging measures and clearly defined clinical outcomes were common strengths. Recurrent limitations included incomplete control of confounding, selection of complete-case samples, limited reporting of follow-up, uncertain temporality, small samples in several organ-specific studies, cohort overlap and lack of external validation. Trial-derived prognostic analyses were not treated as randomised comparisons, whereas the PACMAN-HF nutritional intervention was treated as randomised evidence. Mixed-design studies were interpreted component-wise. The study-to-tool map, report-specific appraisal rationale and principal methodological concerns are provided in Supplementary Table S3; this is not presented as a reconstructed item-by-item historical JBI checklist. No report was excluded solely on the basis of appraisal.

Because several publications contained more than one analytic component and the design-specific appraisal frameworks contain different criteria, item-level judgements were not pooled into a single numerical total or global quality score. The earlier aggregate JBI totals could not be reproduced reliably from the retained study-level audit trail and were therefore removed rather than reconstructed. The appraisal was used to temper domain-level conclusions: hepatic/splanchnic evidence was downgraded interpretively for overlap, confounding and limited external validation; intestinal evidence for temporality and small samples; pancreatic evidence for sparse direct testing and cohort overlap; microbiota and biliary evidence for single-report dependence; nutrition/sarcopenia evidence for non-specificity and complete-case selection; and VExUS evidence for single-cohort external-validation limitations. These are interpretive considerations, not formal certainty grades. Table 3 summarises the design-specific appraisal framework used after reclassification of the actual analysis question.

## 4. Discussion

### 4.1. Principal Findings

The principal finding is not that every digestive organ is equally affected or equally measurable in HF. Rather, the evidence describes a hierarchy. Hepatic biochemical and venous-congestion phenotypes are repeatedly associated with outcomes and have the greatest clinical maturity, although several imaging reports arise from one overlapping cohort programme and external validation remains limited. Intestinal bowel-wall thickening and reduced response to oral loop diuretics provide a biologically plausible mechanistic link, but the key ultrasonographic assessment occurred after initial treatment and therefore does not establish temporal precedence, impaired absorption as the causal mechanism, or a treatment-selection rule. Direct pancreatic exocrine-insufficiency evidence is limited, while circulating pancreatic-enzyme abnormalities, microbiota disturbance, gallbladder-wall thickening and selected gut-barrier biomarkers remain insufficiently replicated. Nutritional decline is clinically important but constitutes a secondary, non-specific expression of interacting cardiac, digestive and systemic pathways.

### 4.2. Interpretation of the Cardio-Gastrointestinal Syndrome Concept

The CGIS concept is therefore useful only if it preserves this gradient of evidence. It should not be presented as an established diagnosis, syndrome with fixed criteria, validated clinical phenotype, or treatment-selection framework. Its current value is conceptual and hypothesis-generating: it connects organ-specific observations that share plausible upstream mechanisms and defines a testable construct for future prospective derivation and external validation. Whether combined phenotypes improve risk stratification, diagnostic performance or treatment selection remains unknown. Candidate phenotypes are summarised in Table 4 solely as an organisational framework and should not be interpreted as validated subtypes, diagnostic categories or guideline-endorsed pathways.

### 4.3. Diagnostic and Prognostic Implications

Routine liver biochemistry remains the most established component of digestive-system assessment in HF, particularly during acute decompensation or right-sided congestion. Bilirubin and cholestatic markers showed the most consistent prognostic associations, whereas aminotransferase findings were less uniform. These associations do not by themselves establish added prognostic value beyond validated HF models. Albumin and selected composite scores may contribute to risk characterization, but MELD- and MELD-XI-based scores also incorporate renal function and should not be interpreted as liver-specific or as substitutes for established HF prognostic tools. Serial change may reflect decongestion, but evidence that serial testing improves clinical decision-making remains limited.

Portal and hepatic venous Doppler, liver stiffness, VExUS and gastrointestinal ultrasonography provide physiological information on systemic and splanchnic congestion. Current studies mainly demonstrate association with haemodynamics or outcomes; only selected reports examined incremental model performance, and few addressed calibration, external validation or diagnostic accuracy against a prespecified reference standard. Prognostic association should therefore not be equated with validated diagnostic utility. Standardised acquisition, operator training, reproducibility studies, independent replication, calibration and clinically actionable thresholds remain prerequisites for wider adoption.

Gut permeability markers, faecal calprotectin, breath tests, microbiota profiles, TMAO and circulating pancreatic enzymes should remain investigational. Their biological plausibility and statistical association are not equivalent to diagnostic accuracy, incremental prognostic value or clinical utility, and serum pancreatic-enzyme concentrations should not be used as a substitute for direct assessment of exocrine insufficiency. Before routine use, candidate markers require reproducible thresholds, appropriate discrimination and calibration analyses where relevant, independent external validation, and evidence that their use changes a clinically meaningful decision or outcome.

### 4.4. Therapeutic Implications

The association between bowel-wall thickening and reduced oral loop-diuretic response is clinically relevant but should not be treated as an actionable CGIS rule. Because intestinal ultrasonography was performed after initial HF treatment, the study does not demonstrate that bowel-wall oedema preceded diuretic non-response or that impaired absorption was the causal mechanism. In a persistently congested patient with poor response to oral therapy, standard causes of apparent diuretic resistance should be assessed according to usual HF care. Decisions regarding intravenous therapy should be based on established clinical indications rather than on the unvalidated bowel-wall finding alone.

**Figure 3:**
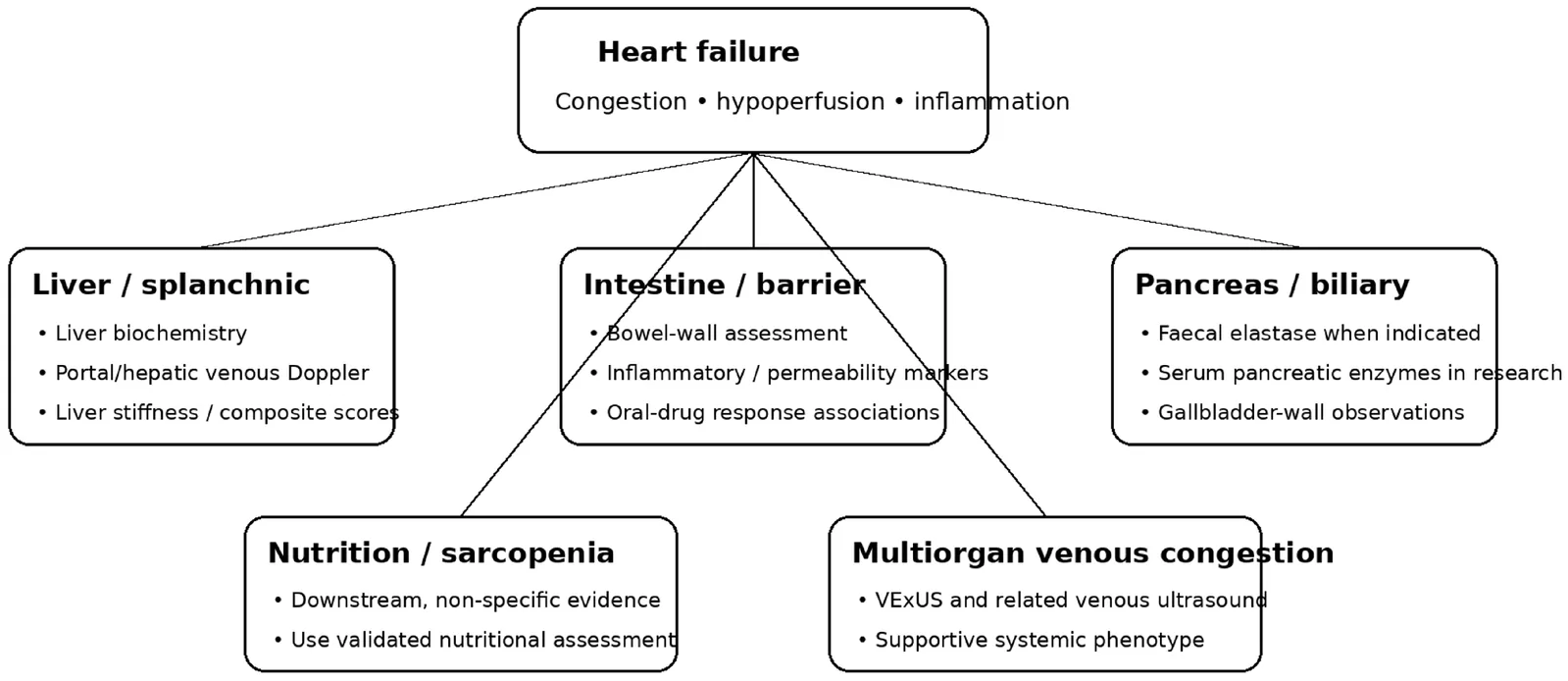
Unvalidated conceptual reasoning framework for digestive-system involvement in heart failure. The figure is hypothesis-generating and is intended to organise research questions and clinical reasoning only; it is not a diagnostic algorithm, guideline pathway, validated phenotype definition or treatment-selection framework.

Nutritional screening and intervention are clinically appropriate in HF independent of the CGIS label. By contrast, pancreatic-enzyme replacement and microbiota-directed therapies cannot be recommended routinely from the present evidence. Future trials should test these interventions only in objectively defined phenotypes, such as confirmed exocrine insufficiency or reproducible barrier/microbial abnormalities. Figure 3 is presented only as an unvalidated conceptual reasoning framework that illustrates research hypotheses and possible domains of assessment; it is not a diagnostic algorithm, management pathway or treatment-selection tool.

### 4.5. Strengths and Limitations

This review has several strengths. It maintains the causal direction from established HF to digestive involvement, separates structured clinical evidence from contextual literature, covers multiple digestive organs and avoids conflating mechanistic plausibility with diagnostic validation. The audited 29-report register permits transparent domain assignment, verifies analysed samples and study designs, and distinguishes publications from approximately 26 underlying cohort families.

Important limitations remain. The evidence base is dominated by observational analyses with heterogeneous HF phenotypes, measurements and follow-up periods. Several domains rely on one or a few reports, and participant overlap could not be quantified precisely within the Fukushima imaging programme or the two Hiki pancreatic-enzyme publications; the 29 reports therefore cannot be treated as 29 independent replications or summed without an overlap check. Nutritional, sarcopenia and VExUS reports provide secondary or multiorgan support rather than digestive-specific evidence. The historical search and selection flow is incompletely auditable at source level: database-specific yields, exact original search dates, the citation-level deduplication trail, reviewer-level historical decisions and the full row-level exclusion register were not recoverable from retained project files. The verified register spans 2016–2025, but no a priori publication-year restriction can be documented; consequently, the absence of older landmark reports from the audited corpus is a limitation of corpus auditability and no claim of exhaustive historical coverage is made. The separate three-database rerun was executed on 8 August 2026 and targeted literature through 1 July 2026, although exact day-level cutoff reproducibility was database-specific and is transparently documented in Supplementary Table S1. Recovery of the ten benchmark reports demonstrates retrievability of those known reports only and does not establish completeness; the 17,051 validation records were not screened as a new or updated corpus. The protocol was not prospectively registered. Risk-of-bias appraisal was necessarily design-specific, and confounding, complete-case selection, uncertain temporality, small samples, cohort overlap and limited external validation constrain inference across several domains. Publication bias could not be assessed meaningfully, and no meta-analysis was justified. More generally, pathway-dependent documentation and biomarker availability can affect real-world gastrointestinal datasets [40]; this reference is used only as a methodological analogy concerning data completeness, not as evidence for HF-related digestive pathology.

### 4.6. Research Priorities

Prospective multi-centre studies should apply simultaneous cardiac, hepatic, intestinal, pancreatic and nutritional measurements across decongestion. Core variables should include right-sided haemodynamics, validated congestion imaging, serial liver tests, bowel-wall assessment, oral drug response and standardised nutritional outcomes. Candidate CGIS phenotypes should be derived and externally validated rather than imposed retrospectively. Studies of microbiota or pancreatic dysfunction should prespecify clinically meaningful thresholds and test whether phenotype-directed intervention improves symptoms, treatment response, hospitalisation or survival.

## 5. Conclusions

Heart failure has clinically relevant digestive consequences that extend beyond the traditional heart–liver axis. Across 29 included reports representing approximately 26 cohort families, hepatic biochemical abnormalities and splanchnic venous-congestion phenotypes had the strongest evidence. Bowel-wall thickening was associated with reduced response to oral loop diuretics, but the post-treatment timing of ultrasonography prevents inference that oedema preceded or caused diuretic non-response. Direct evidence of pancreatic exocrine insufficiency was limited, and microbial, biliary, circulating pancreatic-enzyme and nutritional manifestations remained unevenly replicated, externally unvalidated or non-specific.

CGIS is best presented as a cautious, hypothesis-generating and testable construct that organises these observations without overstating their maturity or independence. It is not a validated syndrome, diagnostic entity, established clinical phenotype or treatment-selection framework. Its clinical value will depend on prospective derivation, independent external validation, standardised measurements and evidence that integrated digestive phenotyping adds information beyond established HF assessment and changes clinically meaningful management or outcomes. Until then, routine liver and nutritional assessment should be distinguished from emerging congestion imaging and investigational gut or pancreatic biomarkers.

## Figures and Tables

**Table 1:** Distribution and maturity of the included clinical reports. Each of the 29 reports is counted once according to its primary domain; secondary multidomain findings are described in the text and do not generate additional report counts.

Domain	Reports, n (Approx. Cohort Families)	Main Measures	Interpretation
Liver/cardio-hepatic and splanchnic	13 (11)	Routine liver tests, MELD/MELD-XI/ALBI-type indices, portal and hepatic venous Doppler	Most mature; repeated but partly overlapping prognostic evidence
Intestinal congestion/barrier/function	5 (5)	Bowel-wall oedema, ultrasound, breath testing, permeability/inflammatory markers	Clinically persuasive but methodologically heterogeneous
Pancreas	4 (3)	Faecal elastase and circulating pancreatic enzymes	Emerging; direct exocrine-insufficiency evidence is limited
Nutrition/sarcopenia/cachexia	4 (4)	Nutritional assessment, sarcopenia and intervention	Secondary/downstream and not digestive-specific
Gut microbiota/intestinal permeability	1 (1)	Microbiota profiling and intestinal permeability	Mechanistic and exploratory
Imaging/VExUS	1 (1)	Multiorgan venous excess ultrasound	Supportive systemic phenotype; not digestive-specific
Biliary/gallbladder congestion	1 (1)	Gallbladder-wall thickness	Preliminary Stage C/D prognostic signal

**Table 2:** Verified characteristics and principal findings of the 29 included reports.

Study	Domain	Verified Design/Type	HF Setting/Analysed Sample	Measurement/Focus	Corrected Principal Finding
Pasini, 2016 [11]	Gut microbiota/intestinal permeability	Cross-sectional case–control observational study	80 total: 60 stable chronic HF + 20 matched controls	Gut microbiota and intestinal permeability	Pathogenic flora and intestinal permeability were higher in HF and correlated with severity, estimated RAP and CRP; not a validated diagnostic microbiota signature.
Valentova, 2016 [12]	Gut barrier/congestion/function	Prospective multi-centre observational cohort	165 chronic HFrEF outpatients; 29 cachectic	Bowel-wall thickness, RV function and cachexia	Bowel-wall thickness was associated with cachexia, GI symptoms, inflammation and higher RAP; the association is not causal.
Mollar, 2019 [13]	Gut barrier/congestion/function	Observational prognostic cohort	102 patients with HF	Hydrogen/methane breath-test AUC	Hydrogen AUC was associated with death/all-cause hospitalisation (HR 1.21 per 1000 U, 95% CI 1.04–1.40) and death/HF hospitalisation (HR 1.20, 95% CI 1.03–1.40); methane AUC was not associated. Breath testing is an indirect SIBO surrogate.
Ikeda, 2021 [14]	Gut barrier/congestion/function	Prospective single-centre observational cohort	213 hospitalised patients with acute HF	Colon-wall thickness and oral/IV diuretic response	Greater colon-wall thickness correlated with higher diuretic dose and poor oral, but not intravenous, loop-diuretic response; ultrasound followed initial treatment, limiting temporality.
Covino, 2024 [15]	Gut barrier/congestion/function	Prospective longitudinal single-centre observational study	59 older patients with acute HF	Faecal calprotectin and zonulin	Faecal calprotectin >50 µg/g, but not serum/faecal zonulin, was independently associated with 90-day death or AHF rehospitalisation; this small single-centre study requires validation.
Hao, 2024 [16]	Gut barrier/congestion/function	Cross-sectional case–control imaging study	82 total: 41 acute HF + 41 healthy controls	GI ultrasonography, motility and venous measures	AHF patients had thicker GI walls, reduced motility and altered splanchnic/hepatic venous measures; no prospective threshold or prognostic validation.
Biegus, 2016 [17]	Liver/cardio-hepatic and splanchnic	Secondary/post-hoc prognostic analysis of PROTECT trial cohort	2033 patients with acute HF	Admission and serial LFTs in PROTECT	Abnormal and worsening LFTs were common and associated with in-hospital and 180-day outcomes. Parent randomisation does not make this analysis an RCT.
Samsky, 2016 [18]	Liver/cardio-hepatic and splanchnic	Secondary prognostic analysis of ASCEND-HF trial data	4228 with complete admission LFT data	Admission LFTs in ASCEND-HF	Only bilirubin retained independent prognostic associations; AST and ALT were not independently associated with outcomes.
Bouabdallaoui, 2020 [19]	Liver/cardio-hepatic and splanchnic	Prospective single-centre observational cohort	95 patients with ADHF	Portal vein pulsatility and EVEREST risk	Portal pulsatility was associated with RV dysfunction and mortality, but added little discrimination to EVEREST (C-index 0.813 to 0.818).
Yoshihisa, 2020 [20]	Liver/cardio-hepatic and splanchnic	Prospective single-centre observational cohort	379 screened; 350 enrolled; analytic N: SWE 342, celiac PSV 321, combined 313	Liver stiffness and coeliac artery peak systolic velocity	High liver stiffness and low celiac PSV were associated with haemodynamics and adverse events.
Sugawara, 2021 [21]	Liver/cardio-hepatic and splanchnic	Prospective single-centre observational cohort	645 screened; 388 underwent ultrasound; 350 analysed	Hepatic-vein waveform patterns	Reversed V-wave pattern was associated with higher RAP and cardiac events (adjusted HR 4.90, 95% CI 2.23–10.74); the cohort belongs to the overlapping Fukushima programme.
Miyama, 2021 [22]	Liver/cardio-hepatic and splanchnic	Prospective multi-centre registry cohort	1158 discharged patients with acute HF	Discharge LFT panels	Elevation of at least two LFT panels at discharge, but not borderline single-panel abnormalities, was associated with death/HF readmission.
Wang, 2021 [23]	Liver/cardio-hepatic and splanchnic	Retrospective large-database cohort	30,096 hospitalised patients with HFpEF	MELD-XI	MELD-XI was independently associated with 60-day all-cause and cardiovascular mortality and 30-day HF readmission. The 2021 corrigendum corrected the title and affiliations and did not alter the scientific conclusions or cohort denominator.
Liang, 2021 [24]	Liver/cardio-hepatic and splanchnic	Post-hoc prognostic analysis of TOPCAT participants	1657 HFpEF participants without chronic hepatic disease	Bilirubin, ALP, AST and ALT in TOPCAT HFpEF	Bilirubin and ALP, but not AST or ALT, were independently associated with outcomes.
Lin, 2022 [25]	Liver/cardio-hepatic and splanchnic	Retrospective single-centre cohort	400 hospitalised patients with chronic HF	MELD-XI	Higher MELD-XI was associated with adjusted 3-year all-cause mortality; MELD-XI is a cardio-hepatic–renal, not liver-specific, score.
Ohara, 2023 [26]	Liver/cardio-hepatic and splanchnic	Prospective single-centre observational cohort	556 underwent ultrasound; 513 enrolled; RHC available in 310	Hepatic venous stasis index	Higher HVSI tracked RAP/right-sided congestion and was associated with cardiac death or worsening HF; external validation is lacking.
Ali, 2025 [27]	Liver/cardio-hepatic and splanchnic	Prospective single-centre ICU observational cohort	164 enrolled; 107 analysed after exclusions	ALBI, MELD-XI, MELD-Alb, FIB-4 and FIB-5	ALBI, MELD-Alb, FIB-4 and FIB-5 were significant as continuous variables after adjustment; MELD-XI was not. ALBI had the highest AUC (0.935).
Curcio, 2025 [28]	Liver/cardio-hepatic and splanchnic	Prospective longitudinal single-centre cohort	162 patients with advanced HF	MELD score	MELD was associated with advanced-HF severity and adverse composite outcomes; it includes renal function and is not a pure hepatic measure.
Myhre, 2025 [29]	Liver/cardio-hepatic and splanchnic	Secondary longitudinal analysis of STRONG-HF	1062 acute HF patients with predischarge LFTs	Serial LFT changes during decongestion	Lower ALT was associated with congestion/lower BMI; LFT reductions tracked decongestion. Baseline ALT had only a non-significant inverse outcome association and LFTs did not modify treatment effect.
Xia, 2017 [30]	Pancreas	Clinical observational study with an enzyme-replacement intervention subgroup	104 chronic HF patients	Faecal elastase-1 and supplementation subgroup	56.7% had FE-1 < 200 µg/g; lower FE-1 correlated with appetite loss/HF severity, and supplementation improved appetite score in a selected subgroup.
Vijver, 2024 [31]	Pancreas	Secondary repeated-measures analysis of PUSH-AHF	274 patients with acute HF	Serial serum amylase and lipase	Amylase and lipase rose modestly during admission and changes correlated with NT-proBNP; baseline values were generally normal. This is not a direct PEI test.
Hiki, J. Clin. Med., 2025 [32]	Pancreas	Single-centre observational prognostic cohort	229 ADHF admissions screened; 146 analysed	Admission pancreatic enzymes and long-term outcomes	Low lipase, but not low amylase, was independently associated with death or ADHF readmission (adjusted HR 1.96, 95% CI 1.16–3.32). Serum enzymes do not establish PEI.
Hiki, Rev. Cardiovasc. Med., 2025 [33]	Pancreas	Single-centre observational case–control study with longitudinal subset	155 ADHF +46 controls enrolled; ADHF regression N = 114; matched comparison 40 + 20; serial subset N = 60	Pancreatic enzymes versus controls and serial change	ADHF patients had lower enzymes than controls; levels correlated with congestion/nutritional parameters and increased after improvement. Enzymes are only suggestive of PEI.
Sakamoto, 2022 [34]	Biliary/gallbladder congestion	Prospective observational imaging cohort with controls	116 enrolled; 86 analysed +11 controls. Of 86: Stage B = 22, Stage C = 60, Stage D = 4	Gallbladder-wall thickness	GB wall thickness increased with stage/congestion; greater GBWT was associated with HF hospitalisation among Stage C/D patients. This remains a single-report signal.
Pagnesi, 2024 [35]	Nutrition/sarcopenia/cachexia	Retrospective multi-centre registry cohort	510 analysed from 1149 HELP-HF patients	GNRI-defined malnutrition	GNRI ≤ 98 occurred in 35.1% and was independently associated with mortality (adjusted HR 2.03, 95% CI 1.43–2.89). No objective digestive-organ measure; 55.6% of the parent registry lacked GNRI data.
López Guillén, 2024 [36]	Nutrition/sarcopenia/cachexia	Cross-sectional single-centre observational study	121 HF outpatients	Nutritional assessment, sarcopenia and frailty	Malnutrition/cachexia and sarcopenia risk were common, but the study contains no direct digestive-system measurement.
Armentaro, 2025 [37]	Nutrition/sarcopenia/cachexia	Retrospective cohort from two registries	670 chronic HF outpatients; 340 sarcopenic and 330 non-sarcopenic	Sarcopenia and prognosis	Sarcopenia was strongly associated with MACE and mortality, but the study deliberately examined well-nourished patients and has no direct digestive outcome.
Anastasiou, 2024 [38]	Imaging/VExUS congestion	Prospective observational imaging cohort	290 patients admitted with acute HF	VExUS score	VexUS 3 was associated with in-hospital mortality (OR 8.03, 95% CI 2.25–28.61); adding VExUS improved model fit over GWTG-HF (Δχ^2^ + 8.44, *p* = 0.03). This is a systemic score, not a digestive-organ test, and lacks external validation.
Ortiz-Cortés, 2025 [39]	Nutrition/sarcopenia/cachexia	Prespecified sub-analysis of the randomised PACMAN-HF trial	225 screened; 64 randomised (31 intervention, 33 control); 59 completed	Nutritional intervention	Intervention improved nutrition and 6MWT but did not significantly reduce death/HF hospitalisation (HR 0.34, 95% CI 0.11–1.09; *p* = 0.072).

Note: Yoshihisa et al., Sugawara et al. and Ohara et al. were treated as linked publications from an overlapping Fukushima cohort programme and therefore as complementary analyses rather than three independent replications. The two Hiki publications were treated as linked reports from a substantially overlapping ADHF cohort. Accordingly, the 29 reports represent approximately 26 cohort families and should not be interpreted as 29 independent replications. Where the degree of participant sharing could not be quantified, overlap was treated conservatively in independence-sensitive interpretation.

**Table 3:** Design-specific appraisal framework after reclassification of the actual analysis question.

Analytic Category	Reports/ Components	JBI Tool	Main Strengths	Recurrent Limitations	Interpretive Rule
Longitudinal/prognostic cohort analyses	Most longitudinal reports, including trial-derived secondary analyses	JBI cohort tool	Objective biomarkers or imaging and defined clinical outcomes	Confounding, complete-case selection, follow-up and temporality	Parent-trial randomisation does not convert a prognostic exposure analysis into an RCT.
Cross-sectional/case-control analyses	Pasini, Hao, López Guillén and the cross-sectional Hiki component	JBI analytical cross-sectional tool	Objective measurements and explicit comparison groups	Selection, group comparability and inability to establish temporality	Associations should not be interpreted causally.
Mixed-design pancreatic report	Xia 2017	Component-wise appraisal	Direct faecal elastase-1 assessment	Small selected supplementation subgroup and incompletely reported intervention safeguards	The observational and intervention components require separate interpretation.
Randomised nutritional intervention	Ortiz-Cortés, 2025 [39]	JBI randomised controlled trial tool	Prespecified intervention comparison	Modest sample, incomplete blinding and limited power for clinical events	Provides downstream nutritional evidence, not a digestive-specific intervention effect.

**Table 4:** Hypothesis-generating cardio-gastrointestinal phenotype framework and current evidentiary status.

Candidate Phenotype	Dominant Mechanism/Context	Candidate Manifestations	Assessment Approach	Current Status
Phenotype 1: acute ischaemic–congestive	Acute decompensation; low flow plus venous congestion	Liver-test changes, intestinal oedema, pancreatic enzyme abnormalities	Routine liver tests; targeted ultrasound; pancreatic enzymes only in research	Hypothesis-generating phenotype
Phenotype 2: chronic congestive–malabsorptive	Persistent right-sided/splanchnic congestion	Bowel-wall oedema, impaired absorption, anorexia and cachexia	Symptoms, nutritional screening, portal/hepatic venous Doppler where available	Hypothesis-generating; unvalidated
Phenotype 3: inflammatory–microbial	Barrier dysfunction, dysbiosis and microbial products	Permeability/inflammatory markers and altered microbiota	No routine biomarker standard	Investigational
Phenotype 4: drug absorption/iatrogenic	Congestion-related impaired oral absorption	Poor response to oral loop diuretics	Clinical response plus congestion assessment	Hypothesis-generating; requires prospective testing
Phenotype 5: nutritional–catabolic	Anorexia, malabsorption, inflammation and catabolism	Malnutrition, sarcopenia and frailty	Validated nutritional and functional assessment	Clinically relevant but not digestive-specific

## Data Availability

No new data were created in this study. The data supporting this review are derived from previously published articles, which are cited throughout the manuscript.

## Supplementary Materials

The following supporting information can be downloaded at: https://www.mdpi.com/article/10.3390/diagnostics16203342/s1, Table S1: Search audit and reproducibility-validation strategies; Table S2: Study–report linkage and overlapping-cohort register; Table S3: Study-level extraction and design-specific methodological-appraisal map.
