# Supplementary material for: Heart Failure Beyond the Heart: Digestive-System Involvement and Its Diagnostic and Prognostic Implications—A Systematic Review
Source: Diagnostics (Basel). 2026 Oct 14;16(20):3342. doi: 10.3390/diagnostics16203342 (PMC13702866; doi:10.3390/diagnostics16203342)
Supplement: Supplementary file 1 [file diagnostics-16-03342-s001.zip › diagnostics-4577549-supplementary.pdf]

Supplementary Materials

Heart Failure Beyond the Heart: Digestive-System Involvement and Its Diagnostic and Prognostic Implications - A Systematic Review

These supplementary tables document the final audit and reproducibility-validation work performed during manuscript closure. The August 2026 validation searches were not used to redefine the historical included corpus and are not interpreted as evidence of exhaustive retrieval.

Table S1. Search audit and reproducibility-validation strategies

Source	Role in final manuscript	Search date	Coverage	Raw yield	Benchmark/reference-set recovery	Audit note
Historical aggregate register	Original study-selection record	Historical; exact database dates unavailable	Not reconstructable source-wise	815 before deduplication	Not applicable	315 duplicates, 500 screened, 150 full text; source-wise yields and citation-level deduplication trail not recoverable.
PubMed/MEDLINE	2026 reproducibility validation	8 Aug 2026	Inception–1 Jul 2026; date cutoff encoded explicitly in the PubMed query	3,660	10/10	Exact query preserved in PubMed history CSV; validation yield not used for study selection.
Scopus	2026 reproducibility validation	8 Aug 2026	Target cutoff 1 Jul 2026; displayed query used PUBYEAR < 2027 and therefore did not encode an exact day-level cutoff	9,122	10/10	RIS export archived. The 9,122-record yield is a reproducibility-validation yield only. Because the displayed syntax uses a year filter rather than an exact date limit, day-level coverage through 1 Jul 2026 cannot be independently demonstrated from the query text alone.
Web of Science Core Collection	2026 reproducibility validation	8 Aug 2026	Target cutoff 1 Jul 2026; the retained audit records this cutoff, although the displayed TS syntax does not itself encode a date limit	4,269	10/10	Five RIS batches archived; validation yield not used for study selection. The exact day-level cutoff is documented in the audit record

Source	Role in final manuscript	Search date	Coverage	Raw yield	Benchmark/reference-set recovery	Audit note
						but is not reproducible from the displayed TS syntax alone.
Embase	Not included in 2026 validation rerun	Not run	—	—	—	Access unavailable for the 2026 validation rerun; no database-specific historical Embase yield or reconstructed validation result is asserted.

S1A. PubMed/MEDLINE validation strategy

```
( "Heart Failure"[Mesh] OR "heart failure"[tiab] OR "cardiac failure"[tiab] OR "congestive heart failure"[tiab] OR "acute heart failure"[tiab] OR "chronic heart failure"[tiab] OR "acute decompensated heart failure"[tiab] OR "decompensated heart failure"[tiab] OR HFrEF[tiab] OR HFpEF[tiab] OR HFmrEF[tiab] OR "right heart failure"[tiab] OR "right-sided heart failure"[tiab] ) AND ( "Liver Function Tests"[Mesh] OR "Liver Circulation"[Mesh] OR "Portal System"[Mesh] OR "Splanchnic Circulation"[Mesh] OR "Gastrointestinal Absorption"[Mesh] OR "Gastrointestinal Microbiome"[Mesh] OR "Exocrine Pancreatic Insufficiency"[Mesh] OR "Pancreatic Function Tests"[Mesh] OR "intestinal congestion"[tiab] OR "intestinal edema"[tiab] OR "intestinal oedema"[tiab] OR "bowel wall edema"[tiab] OR "bowel wall oedema"[tiab] OR "intestinal wall thickening"[tiab] OR "gastrointestinal wall thickening"[tiab] OR "intestinal function"[tiab] OR "gastrointestinal function"[tiab] OR "intestinal blood flow"[tiab] OR "splanchnic blood flow"[tiab] OR "gastrointestinal absorption"[tiab] OR "intestinal absorption"[tiab] OR malabsor*[tiab] OR "gastrointestinal motility"[tiab] OR "intestinal motility"[tiab] OR "intestinal permeability"[tiab] OR "gut permeability"[tiab] OR "intestinal barrier"[tiab] OR "gut barrier"[tiab] OR "intestinal inflammation"[tiab] OR calprotectin[tiab] OR endotoxin*[tiab] OR "bacterial translocation"[tiab] OR microbiot*[tiab] OR microbiom*[tiab] OR dysbios*[tiab] OR "gut flora"[tiab] OR TMAO[tiab] OR "trimethylamine N-oxide"[tiab] OR SIBO[tiab] OR "small intestinal bacterial overgrowth"[tiab] OR "breath test*"[tiab] OR "liver function test*"[tiab] OR "liver dysfunction"[tiab] OR "hepatic dysfunction"[tiab] OR "hepatic congestion"[tiab] OR "liver congestion"[tiab] OR "congestive hepatopathy"[tiab] OR "cardiac hepatopathy"[tiab] OR "hypoxic hepatitis"[tiab] OR "ischemic hepatitis"[tiab] OR "hepatic hemodynamic*"[tiab] OR "hepatic haemodynamic*"[tiab] OR "hepatic vein"[tiab] OR "hepatic venous"[tiab] OR "portal vein"[tiab] OR "portal venous"[tiab] OR "portal doppler"[tiab] OR splanchnic[tiab] OR MELD[tiab] OR "MELD-XI"[tiab] OR ALBI[tiab] OR "hepatic venous stasis index"[tiab:~0] OR "pancreatic exocrine insufficiency"[tiab] OR "exocrine pancreatic insufficiency"[tiab] OR "pancreatic dysfunction"[tiab] OR "pancreatic function"[tiab] OR "pancreatic enzyme*"[tiab] OR "faecal elastase"[tiab] OR "fecal elastase"[tiab] OR "gallbladder wall"[tiab] OR "gallbladder congestion"[tiab:~0] OR "biliary congestion"[tiab:~0] OR "gastrointestinal ultrasound"[tiab] OR "gastrointestinal ultrasonograph*"[tiab] OR VExUS[tiab] OR "venous excess ultrasound"[tiab] OR cardiointestin*[tiab] OR "cardio-gastrointestinal"[tiab] ) AND 1800/01/01:2026/07/01[dp]
```

S1B. Scopus validation strategy

```
TITLE-ABS-KEY(
"heart failure" OR "cardiac failure" OR "congestive heart failure" OR "acute heart failure" OR "chronic heart failure" OR "acute decompensated heart failure" OR "decompensated heart failure" OR HFrEF OR HFpEF OR HFmrEF OR "right heart failure" OR "right-sided heart failure")
AND TITLE-ABS-KEY(
"intestinal congestion" OR "intestinal edema" OR "intestinal oedema" OR "bowel wall edema" OR "bowel wall oedema" OR "intestinal wall thickening" OR "gastrointestinal wall thickening" OR "intestinal function" OR "gastrointestinal function" OR "intestinal blood flow" OR "splanchnic blood flow" OR "gastrointestinal absorption" OR "intestinal absorption" OR malabsor* OR "gastrointestinal motility" OR "intestinal motility" OR "intestinal permeability" OR "gut permeability" OR "intestinal barrier" OR "gut barrier" OR "intestinal inflammation" OR calprotectin OR endotoxin* OR "bacterial translocation" OR microbiot* OR microbiom* OR dysbios* OR "gut flora" OR TMAO OR "trimethylamine N-oxide" OR SIBO OR "small intestinal bacterial overgrowth" OR "breath test*" OR "liver function test*" OR "liver dysfunction" OR "hepatic dysfunction" OR "hepatic congestion" OR "liver congestion" OR "congestive hepatopathy" OR "cardiac hepatopathy" OR "hypoxic hepatitis" OR "ischemic hepatitis" OR "hepatic hemodynamic*" OR "hepatic haemodynamic*" OR "hepatic vein" OR "hepatic venous" OR "portal vein" OR "portal venous" OR "portal doppler" OR splanchnic OR MELD OR "MELD-XI" OR ALBI OR "hepatic venous stasis index" OR "pancreatic exocrine insufficiency" OR "exocrine pancreatic insufficiency" OR "pancreatic dysfunction" OR "pancreatic function" OR "pancreatic enzyme*" OR "faecal elastase" OR "fecal elastase" OR "gallbladder wall" OR "gallbladder congestion" OR "biliary congestion" OR "gastrointestinal ultrasound" OR "gastrointestinal ultrasonograph*" OR VExUS OR "venous excess ultrasound" OR cardiointestin* OR "cardio-gastrointestinal")
AND PUBYEAR < 2027
```

S1C. Web of Science Core Collection validation strategy

```
TS=(
"heart failure" OR "cardiac failure" OR "congestive heart failure" OR "acute heart failure" OR "chronic heart failure" OR
"acute decompensated heart failure" OR "decompensated heart failure" OR HFrEF OR HFpEF OR HFmrEF OR "right heart failure" OR
"right-sided heart failure")
AND TS=(
"intestinal congestion" OR "intestinal edema" OR "intestinal oedema" OR "bowel wall edema" OR "bowel wall oedema" OR
"intestinal wall thickening" OR "gastrointestinal wall thickening" OR "intestinal function" OR "gastrointestinal function" OR
"intestinal blood flow" OR "splanchnic blood flow" OR "gastrointestinal absorption" OR "intestinal absorption" OR malabsor* OR
"gastrointestinal motility" OR "intestinal motility" OR "intestinal permeability" OR "gut permeability" OR "intestinal
barrier" OR "gut barrier" OR "intestinal inflammation" OR calprotectin OR endotoxin* OR "bacterial translocation" OR
microbiot* OR microbiom* OR dysbios* OR "gut flora" OR TMAO OR "trimethylamine N-oxide" OR SIBO OR "small intestinal bacterial
overgrowth" OR "breath test*" OR "liver function test*" OR "liver dysfunction" OR "hepatic dysfunction" OR "hepatic
congestion" OR "liver congestion" OR "congestive hepatopathy" OR "cardiac hepatopathy" OR "hypoxic hepatitis" OR "ischemic
hepatitis" OR "hepatic hemodynamic*" OR "hepatic haemodynamic*" OR "hepatic vein" OR "hepatic venous" OR "portal vein" OR
"portal venous" OR "portal doppler" OR splanchnic OR MELD OR "MELD-XI" OR ALBI OR "hepatic venous stasis index" OR "pancreatic
exocrine insufficiency" OR "exocrine pancreatic insufficiency" OR "pancreatic dysfunction" OR "pancreatic function" OR
"pancreatic enzyme*" OR "faecal elastase" OR "fecal elastase" OR "gallbladder wall" OR "gallbladder congestion" OR "biliary
congestion" OR "gastrointestinal ultrasound" OR "gastrointestinal ultrasonograph*" OR VExUS OR "venous excess ultrasound" OR
cardiointestin* OR "cardio-gastrointestinal")
```

Search-date note. The validation rerun was executed on 8 August 2026. The intended evidence cutoff was 1 July 2026. PubMed encoded this cutoff directly in the query. For Scopus and Web of Science, the retained materials do not permit the same day-level cutoff to be reproduced from the displayed search syntax alone. This limitation is reported transparently and does not affect the historical 29-report corpus, because the validation searches were not used for study selection.

Table S2. Study–report linkage and overlapping-cohort register

Ref.	Report	Domain	Cohort family ID	Independence status	Overlap basis	Interpretive rule
11	Pasini et al., 2016	Microbiota / intestinal permeability	FAM-11	Independent within included set	No overlap identified within the included set.	Count as one report and one cohort family.
12	Valentova et al., 2016	Intestinal congestion / cachexia	FAM-12	Independent within included set	No overlap identified.	Count as one report and one cohort family.
13	Mollar et al., 2019	Breath testing / putative SIBO	FAM-13	Independent within included set	No overlap identified.	Count as one report and one cohort family.
14	Ikeda et al., 2021	Intestinal oedema / oral diuretic response	FAM-14	Independent within included set	No overlap identified.	Count as one report and one cohort family.
15	Covino et al., 2024	Intestinal inflammation / permeability	FAM-15	Independent within included set	No overlap identified.	Count as one report and one cohort family.
16	Hao et al., 2024	Gastrointestinal ultrasonography / motility	FAM-16	Independent within included set	No overlap identified.	Count as one report and one cohort family.
17	Biegus et al., 2016	Liver tests in acute HF	FAM-17	Independent within included set	No overlap identified.	Count as one report and one cohort family.
18	Samsky et al., 2016	Liver tests in acute HF	FAM-18	Independent within included set	No overlap identified.	Count as one report and one cohort family.
19	Bouabdallaoui et al., 2020	Portal venous Doppler	FAM-19	Independent within included set	No overlap identified.	Count as one report and one cohort family.
20	Yoshihisa et al., 2020	Hepatic congestion and hypoperfusion imaging	FUKUSHIMA-IMAGING	Linked/overlapping	Near-certain nested Fukushima cohort family with Sugawara 2021 and Ohara 2023.	Retain report-specific outcome; count one cohort family for independence-sensitive interpretation.
21	Sugawara et al., 2021	Hepatic vein Doppler	FUKUSHIMA-IMAGING	Linked/overlapping	Near-certain nested Fukushima cohort family with Yoshihisa 2020 and Ohara 2023.	Retain report-specific outcome; count one cohort family for independence-sensitive interpretation.

Ref.	Report	Domain	Cohort family ID	Independence status	Overlap basis	Interpretive rule
22	Miyama et al., 2021	Liver tests in acute HF	FAM-22	Independent within included set	No overlap identified.	Count as one report and one cohort family.
23	Wang et al., 2021	MELD-XI in HFpEF	FAM-23	Independent within included set	No overlap identified.	Count as one report and one cohort family.
24	Liang et al., 2021	Liver tests in HFpEF	FAM-24	Independent within included set	No overlap identified.	Count as one report and one cohort family.
25	Lin et al., 2022	MELD-XI in chronic HF	FAM-25	Independent within included set	No overlap identified.	Count as one report and one cohort family.
26	Ohara et al., 2023	Hepatic venous stasis index	FUKUSHIMA-IMAGING	Linked/overlapping	Near-certain nested Fukushima cohort family with Yoshihisa 2020 and Sugawara 2021.	Retain report-specific outcome; count one cohort family for independence-sensitive interpretation.
27	Ali et al., 2025	Composite liver dysfunction indices	FAM-27	Independent within included set	No overlap identified.	Count as one report and one cohort family.
28	Curcio et al., 2025	MELD in advanced HF	FAM-28	Independent within included set	No overlap identified.	Count as one report and one cohort family.
29	Myhre et al., 2025	LFT dynamics in STRONG-HF	FAM-29	Independent within included set	No overlap identified.	Count as one report and one cohort family.
30	Xia et al., 2017	Pancreatic exocrine insufficiency	FAM-30	Independent within included set	No overlap identified.	Count as one report and one cohort family.
31	Vijver et al., 2024	Pancreatic enzyme dynamics	FAM-31	Independent within included set	No overlap identified.	Count as one report and one cohort family.
32	Hiki et al., J Clin Med 2025	Pancreatic enzymes and prognosis	JUNTENDO-PANCREAS	Linked/overlapping	Definite substantial overlap with Hiki et al., Rev Cardiovasc Med 2025.	Retain report-specific outcome; count one cohort family for independence-sensitive interpretation.
33	Hiki et al., Rev Cardiovasc Med 2025	Pancreatic enzyme phenotype	JUNTENDO-PANCREAS	Linked/overlapping	Definite substantial overlap with the J Clin Med prognostic report.	Retain report-specific outcome; count one cohort family for independence-sensitive interpretation.
34	Sakamoto et al., 2022	Gallbladder-wall congestion	FAM-34	Independent within included set	No overlap identified.	Count as one report and one cohort family.
35	Pagnesi et al., 2024	Malnutrition in severe HF	FAM-35	Independent within included set	No overlap identified.	Count as one report and one cohort family.
36	López Guillén et al., 2024	Nutritional status / sarcopenia	FAM-36	Independent within included set	No overlap identified.	Count as one report and one cohort family.
37	Armentaro et al., 2025	Sarcopenia	FAM-37	Independent within included set	No overlap identified.	Count as one report and one cohort family.
38	Anastasiou et al., 2024	VExUS multiorgan congestion	FAM-38	Independent within included set	No overlap identified.	Count as one report and one cohort family.
39	Ortiz-Cortés et al., 2025	Nutritional intervention	FAM-39	Independent within included set	No overlap identified.	Count as one report and one cohort family.

Overlap-classification note. Cohort overlap was assessed using centre/institution, investigators/authorship, recruitment interval, baseline sample size, HF phenotype, inclusion criteria, participant characteristics and parent study programme, trial or registry. Complete overlap denotes the same underlying participant cohort; substantial overlap denotes clearly shared participants with non-identical analytic samples; possible overlap denotes plausible shared recruitment that cannot be quantified from the available reports;

and independent cohort denotes no material overlap identified within the included set. Linked reports were retained when they contributed distinct outcomes but were not counted as independent replications in independence-sensitive interpretation.

Table S3. Study-level extraction and design-specific methodological-appraisal map

Ref.	Report	Actual analysis design	Analysed sample	Principal finding	Appraisal framework	Appraisal rationale / principal methodological concern	Evidence stratum	Audit status
11	Pasini et al., 2016	Cross-sectional case–control observational study	80 total: 60 stable chronic HF + 20 matched controls	Pathogenic flora and intestinal permeability were higher in HF and correlated with severity, estimated RAP and CRP; not a validated diagnostic microbiota signature.	JBI analytical cross-sectional tool	Cross-sectional comparison limits temporality and causal inference; modest sample and residual confounding may influence microbiota and permeability associations.	Core direct evidence	Included; interpretation corrected
12	Valentova et al., 2016	Prospective multicentre observational cohort	165 chronic HFrEF outpatients; 29 cachectic	Bowel-wall thickness was associated with cachexia, GI symptoms, inflammation and higher RAP; the association is not causal.	JBI cohort tool / prognostic cohort framework	Observational cohort design supports association but not causality; the cachectic subgroup was small and residual confounding remains possible.	Core direct evidence	Included; interpretation corrected
13	Mollar et al., 2019	Observational prognostic cohort	102 patients with HF	Hydrogen AUC, but not methane AUC, was associated with death/hospitalisation outcomes; breath testing is an indirect SIBO surrogate.	JBI cohort tool / prognostic cohort framework	Modest prognostic cohort with an indirect breath-test surrogate for SIBO; residual confounding and limited external validation constrain clinical interpretation.	Core direct evidence	Included; interpretation corrected
14	Ikeda et al., 2021	Prospective single-centre observational cohort	213 hospitalised patients with acute HF	Greater colon-wall thickness correlated with higher diuretic dose and poor oral, but not intravenous, loop-diuretic response; ultrasound followed initial treatment, limiting temporality.	JBI cohort tool / prognostic cohort framework	Ultrasonography was performed after initial HF treatment, creating uncertain temporality; single-centre observational data cannot establish impaired absorption as the causal mechanism of oral diuretic resistance.	Core direct evidence	Included; interpretation corrected
15	Covino et al., 2024	Prospective longitudinal single-centre observational study	59 older patients with acute HF	Faecal calprotectin >50 µg/g, but not serum/faecal zonulin, predicted 90-day death or AHF rehospitalisation.	JBI cohort tool / prognostic cohort framework	Small, single-centre cohort of older patients; the calprotectin threshold requires independent validation and zonulin findings were negative.	Core direct evidence	Included; interpretation corrected

Ref.	Report	Actual analysis design	Analysed sample	Principal finding	Appraisal framework	Appraisal rationale / principal methodological concern	Evidence stratum	Audit status
16	Hao et al., 2024	Cross-sectional case–control imaging study	82 total: 41 acute HF + 41 healthy controls	AHF patients had thicker GI walls, reduced motility and altered splanchnic/hepatic venous measures; no prospective threshold or prognostic validation.	JBI analytical cross-sectional tool	Cross-sectional case–control design with healthy controls cannot establish temporality or prospective diagnostic/prognostic performance; no externally validated threshold was derived.	Core direct evidence	Included; interpretation corrected
17	Biegus et al., 2016	Secondary/post-hoc prognostic analysis of PROTECT trial cohort	2,033 patients with acute HF	Abnormal and worsening LFTs were common and associated with in-hospital and 180-day outcomes. Parent randomisation does not make this analysis an RCT.	JBI cohort tool / prognostic cohort framework	Secondary prognostic analysis of a parent RCT; the exposure–outcome relationship is observational and remains vulnerable to residual confounding despite randomized treatment assignment in the parent trial.	Core direct evidence	Included; interpretation corrected
18	Samsky et al., 2016	Secondary prognostic analysis of ASCEND-HF trial data	4,228 with complete admission LFT data	Only bilirubin retained independent prognostic associations; AST and ALT were not independently associated with outcomes.	JBI cohort tool / prognostic cohort framework	Secondary complete-case prognostic analysis of trial data; selection related to available liver tests and residual confounding limit causal interpretation.	Core direct evidence	Included; interpretation corrected
19	Bouabdallaoui et al., 2020	Prospective single-centre observational cohort	95 patients with ADHF	Portal pulsatility was associated with RV dysfunction and mortality, but added little discrimination to EVEREST (C-index 0.813 to 0.818).	JBI cohort tool / prognostic cohort framework	Single-centre cohort with modest sample size; portal pulsatility showed only small incremental discrimination beyond the EVEREST score and lacks external validation.	Core direct evidence	Included; interpretation corrected
20	Yoshihisa et al., 2020	Prospective single-centre observational cohort	379 screened; 350 enrolled; analytic N: SWE 342, celiac PSV 321, combined 313	High liver stiffness and low celiac PSV were associated with haemodynamics and adverse events.	JBI cohort tool / prognostic cohort framework	Single-centre imaging cohort with measurement-specific analytic subsets and overlap with later Fukushima reports; findings should be interpreted as complementary rather than independent replication.	Linked report from overlapping cohort	Included; interpretation corrected
21	Sugawara et al., 2021	Prospective single-centre	645 screened; 388	Reversed V-wave pattern was	JBI cohort tool / prognostic cohort	Single-centre imaging	Linked report from overlapping cohort	Included; interpretation

Ref.	Report	Actual analysis design	Analysed sample	Principal finding	Appraisal framework	Appraisal rationale / principal methodological concern	Evidence stratum	Audit status
		observational cohort	underwent ultrasound; 350 analysed	associated with higher RAP and adverse events (adjusted HR 4.90).	framework	cohort overlapping the Fukushima programme; waveform findings require independent external validation despite adjusted prognostic association.		corrected
22	Miyama et al., 2021	Prospective multicentre registry cohort	1,158 discharged patients with acute HF	Elevation of at least two LFT panels at discharge, but not borderline single-panel abnormalities, predicted death/HF readmission.	JBI cohort tool / prognostic cohort framework	Registry analysis restricted to patients discharged alive; residual confounding and discharge-survivor selection may affect prognostic estimates.	Core direct evidence	Included; interpretation corrected
23	Wang et al., 2021	Retrospective large-database cohort	30,096 hospitalised patients with HFpEF	MELD-XI was independently associated with 60-day all-cause mortality, cardiovascular mortality and 30-day HF readmission. The 2021 corrigendum corrected the title and author affiliations and did not alter the scientific conclusions or cohort denominator.	JBI cohort tool / prognostic cohort framework	Retrospective large-database cohort; MELD-XI is a composite hepatic–renal score and associations may be influenced by residual confounding and database-level measurement limitations.	Core direct evidence	Included; bibliographic and interpretation correction resolved
24	Liang et al., 2021	Post-hoc prognostic analysis of TOPCAT participants	1,657 HFpEF participants without chronic hepatic disease	Bilirubin and ALP, but not AST or ALT, were independently associated with outcomes.	JBI cohort tool / prognostic cohort framework	Post-hoc prognostic analysis of TOPCAT participants; trial-population selection and residual confounding limit generalisability beyond the analysed HFpEF cohort.	Core direct evidence	Included; interpretation corrected
25	Lin et al., 2022	Retrospective single-centre cohort	400 hospitalised patients with	Higher MELD-XI was associated with adjusted 3-	JBI cohort tool / prognostic cohort framework	Retrospective single-centre cohort; MELD-	Core direct evidence	Included; interpretation corrected

Ref.	Report	Actual analysis design	Analysed sample	Principal finding	Appraisal framework	Appraisal rationale / principal methodological concern	Evidence stratum	Audit status
			chronic HF	year all-cause mortality; MELD-XI is a cardiohepatic–renal, not liver-specific, score.		XI is not liver-specific and the prognostic association requires external validation in independent settings.		
26	Ohara et al., 2023	Prospective single-centre observational cohort	556 underwent ultrasound; 513 enrolled; RHC available in 310	Higher HVSI tracked RAP/right-sided congestion and predicted cardiac death or worsening HF.	JBI cohort tool / prognostic cohort framework	Single-centre report from the overlapping Fukushima imaging programme; right-heart catheterisation was available only in a subset and the findings are not an independent replication.	Linked report from overlapping cohort	Included; interpretation corrected
27	Ali et al., 2025	Prospective single-centre ICU observational cohort	164 enrolled; 107 analysed after exclusions	ALBI, MELD-Alb, FIB-4 and FIB-5 were significant as continuous variables after adjustment; MELD-XI was not. ALBI had the highest AUC (0.935).	JBI cohort tool / prognostic cohort framework	Only 107 of 164 enrolled ICU patients were analysed; exclusions, modest sample size and absence of external validation may inflate apparent discrimination of composite scores.	Core direct evidence	Included; interpretation corrected
28	Curcio et al., 2025	Prospective longitudinal single-centre cohort	162 patients with advanced HF	MELD was associated with advanced-HF severity and adverse composite outcomes; it includes renal function and is not a pure hepatic measure.	JBI cohort tool / prognostic cohort framework	Single-centre advanced-HF cohort; MELD includes renal function and therefore cannot be interpreted as a liver-specific measure, and external validation is limited.	Core direct evidence	Included; interpretation corrected
29	Myhre et al., 2025	Secondary longitudinal analysis of STRONG-HF	1,062 acute HF patients with predischarge LFTs	Lower ALT was associated with congestion/lower BMI; LFT reductions tracked decongestion. Baseline ALT had only a non-significant inverse outcome association and LFTs did not modify treatment effect.	JBI cohort tool / prognostic cohort framework	Secondary longitudinal analysis of STRONG-HF; liver-test dynamics occurred during active decongestion and treatment, and no significant treatment interaction was demonstrated.	Core direct evidence	Included; interpretation corrected
30	Xia et al., 2017	Clinical observational study with an enzyme-replacement intervention subgroup	104 chronic HF patients	56.7% had FE-1 <200 μg/g; lower FE-1 correlated with appetite loss/HF severity, and supplementation improved appetite score in a selected	Component-wise appraisal: analytical cross-sectional/observational component; intervention subgroup separately	Mixed observational/intervention report; direct faecal-elastase assessment is informative, but the	Core direct evidence	Included; interpretation corrected

Ref .	Report	Actual analysis design	Analysed sample	Principal finding	Appraisal framework	Appraisal rationale / principal methodological concern	Evidence stratum	Audit status
				subgroup.		supplementation subgroup was selected and small, with limited intervention safeguards and no basis for causal treatment inference.		
31	Vijver et al., 2024	Secondary repeated-measures analysis of PUSH-AHF	274 patients with acute HF	Amylase and lipase rose modestly during admission and changes correlated with NT-proBNP; baseline values were generally normal. This is not a direct PEI test.	JBI cohort tool / prognostic cohort framework	Secondary repeated-measures analysis of a parent acute-HF trial; serum enzyme changes are not a direct test of pancreatic exocrine insufficiency and causal mechanisms remain uncertain.	Core direct evidence	Included; interpretation corrected
32	Hiki et al., J Clin Med 2025	Single-centre observational prognostic cohort	229 ADHF admissions screened; 146 analysed	Low lipase, but not low amylase, independently predicted death or ADHF readmission (HR 1.96). Serum enzymes do not establish PEI.	JBI cohort tool / prognostic cohort framework	Single-centre prognostic cohort with substantial overlap with the companion Hiki report; analysed sample was selected from screened admissions and the association lacks independent external validation.	Linked report from overlapping cohort	Included; interpretation corrected
33	Hiki et al., Rev Cardiovasc Med 2025	Single-centre observational case–control study with longitudinal subset	155 ADHF +46 controls enrolled; ADHF regression N=114; matched comparison 40+20; serial subset N=60	ADHF patients had lower enzymes than controls; levels correlated with congestion/nutritional parameters and increased after improvement. Enzymes are only suggestive of PEI.	JBI analytical cross-sectional tool	Substantially overlapping companion report with several different analytic subsets; case–control and longitudinal components are vulnerable to selection and cannot establish pancreatic exocrine insufficiency from serum enzymes.	Linked report from overlapping cohort	Included; interpretation corrected
34	Sakamoto et al., 2022	Prospective observational imaging cohort with controls	116 enrolled; 86 analysed +11 controls. Of 86: Stage B=22, Stage C=60, Stage D=4	GB wall thickness increased with stage/congestion; high GBWT predicted HF hospitalisation among Stage C/D patients.	JBI cohort tool / prognostic cohort framework	Only 86 of 116 enrolled patients were analysed and prognostic interpretation relies on the small Stage C/D subgroup; single-centre findings require	Conditional/borderline evidence	Included; interpretation corrected

Ref.	Report	Actual analysis design	Analysed sample	Principal finding	Appraisal framework	Appraisal rationale / principal methodological concern	Evidence stratum	Audit status
						independent validation.		
35	Pagnesi et al., 2024	Retrospective multicentre registry cohort	510 analysed from 1,149 HELP-HF patients	GNRI ≤98 occurred in 35.1% and independently predicted mortality (adjusted HR 2.03). No objective digestive-organ measure; 55.6% were excluded for missing GNRI data.	JBI cohort tool / prognostic cohort framework	Complete-case analysis included 510 of 1,149 registry patients, with 55.6% excluded for missing GNRI data; substantial missingness may introduce selection bias, and no direct digestive-organ measure was included.	Secondary/downstream CGIS evidence	Included; interpretation corrected
36	López Guillén et al., 2024	Cross-sectional single-centre observational study	121 HF outpatients	Malnutrition/cachexia and sarcopenia risk were common, but the study contains no direct digestive-system measurement.	JBI analytical cross-sectional tool	Cross-sectional single-centre study with no direct digestive-system measurement; temporality cannot be established and findings are appropriately treated as downstream nutritional evidence.	Secondary/downstream CGIS evidence	Included; interpretation corrected
37	Armentaro et al., 2025	Retrospective cohort from two registries	670 chronic HF outpatients; 340 sarcopenic and 330 non-sarcopenic	Sarcopenia strongly predicted MACE and mortality, but the study deliberately examined well-nourished patients and has no direct digestive outcome.	JBI cohort tool / prognostic cohort framework	Retrospective registry analysis intentionally focused on well-nourished patients and included no direct digestive-organ outcome; residual confounding limits attribution to digestive mechanisms.	Secondary/downstream CGIS evidence	Included; interpretation corrected
38	Anastasiou et al., 2024	Prospective observational imaging cohort	290 patients admitted with acute HF	VExUS 3 was associated with in-hospital mortality (OR 8.03) and improved prediction over GWTG-HF. It is a systemic score, not a digestive-organ test.	JBI cohort tool / prognostic cohort framework	Prospective systemic-congestion imaging study, but VExUS is not digestive-organ specific; incremental prognostic findings require external validation and should not be interpreted as diagnostic accuracy for CGIS.	Secondary/downstream CGIS evidence	Included; interpretation corrected
39	Ortiz-Cortés et al., 2025	Prespecified subanalysis of the randomised PACMAN-HF trial	225 screened; 64 randomised (31 intervention, 33 control); 59	Intervention improved nutrition and 6MWT but did not significantly reduce death/HF hospitalisation (HR	JBI randomised controlled trial tool	Small randomized nutritional subanalysis with 64 participants and 59 completers;	Secondary/downstream CGIS evidence	Included; interpretation corrected

Ref.	Report	Actual analysis design	Analysed sample	Principal finding	Appraisal framework	Appraisal rationale / principal methodological concern	Evidence stratum	Audit status
			completed	0.34, p=0.072).		limited power for clinical events and incomplete blinding restrict conclusions about hard outcomes.		

Note: the methodological-appraisal map is intentionally reported at study/component level. Earlier pooled totals across different JBI instruments were withdrawn because the tools contain non-equivalent domains and the preserved project files did not support reproducible reconstruction of the aggregate item counts.

Appraisal note. Table S3 reports the verified study/component-level appraisal mapping used in the revised synthesis: actual analysis design, design-appropriate JBI framework, concise appraisal rationale, principal methodological concern and evidence stratum. It is not an item-by-item reconstruction of historical JBI checklist responses. The preserved files do not support reproducible recreation of those checklist-level entries, and no such judgements have been fabricated retrospectively. The qualitative concern statements are used to constrain interpretation rather than to generate a global numerical quality score.
